# Making Metallic Dies

**Published:** 1855-04

**Authors:** 


### ARTICLE X.

### *Making Metallic Dies.*

Much diversity of opinion exists in the profession as to the best method of getting up metal casts for the purpose of swaging plates, some preferring to immerse the plaster model in molten lead, others to pour led upon the plaster cast, others to cover the wax impression with various coats, principally plumbago, and pour an alloy of metals thereon, which melts at a very low temperature, say 170° Farenheit, but we question much whether there can be an improvement upon the plan most common, but still not universal, as we opine it will be ere long.

This consists of obtaining a plaster model from wax impression, trimming to permit its being withdrawn from the sand, placing this model on a smooth surface, or small board, and using a cast iron wagon box, of sufficient size as to leave a space of an inch between it and the cast; then sieve the finest loom sand around until the cast is covered secure from any rough particles being rammed down upon the model as would spoil the smooth surface of the impression when the plaster cast has been withdrawn, then lightly ram the sand in the box until it is full; this must be done with care, so as not to render the sand so hard and dense as to preclude the passage of the gases and steam generated by the contact of hot metal. In tempering the sand for moulding, I should remark, that it must not be made too wet, for this would be inevitably followed by the bubbling of the metal when poured within from the steam formed. To prevent these accidents, a small wire is frequently used to introduce through the packed sand, in many places, down to the plaster model for the purpose of conducting off the gas and steam, and is altogether a safe preventive; before moulding, the sand should be tested as regards its moisture and adhesiveness which is done by squeezing a handful together, and when it is found to adhere with some firmness, it will answer well to use. To withdraw the model after the box is turned over requires no little art, but which is readily acquired by practice; a small instrument should now be forced into the plaster model and with a light hammer tapped in all directions, so as to slightly enlarge the impression, and relieve the model from any adhering particles; this, of course, is assisted by the smooth coating of varnish put on the plaster cast after it is taken from the wax impression. Some also aid this by shaking over it, from a bag made of very thick and fine woollen cloth, a compound of pulverized soap stone, flour and charcoal, when the model is placed within the box for the sand impression. This composition also gives a polished surface to the metal by filling up the little holes in the sand's surface, and by preventing any particles adhering to the metal.

The rapping spoken of, if done carefully, will, to a great extent, overcome the shrinkage so injurious in many cases found in fitting plates to hard and unyielding palates. It is preferable to withdraw the plaster model, in most cases, from the sand by the instrument instead of throwing it out by a jerk, in reversing the box, from the great liability of the projecting edges of the cast, injuring the surfaces of the sand impression. In fact, in many cases, it cannot be thrown, from the great projection of the alveolar border extending out so far, and can be only withdrawn by tilting the model towards this projection while removing it, and at the same time, as it were, depressing it. The composition of the male die, should be made of $\frac{2}{3}$ zinc and $\frac{1}{3}$ tin, the female of pure lead.

We have never found any difficulty in fitting the most unfavorable mouths with plates struck up between such dies, and thereby are led to recommend such to general use. To take the female die, use a sheet iron plate, with an aperture cut therein, sufficiently large to pass over the male die to the point that the female die should reach, then imbed the former in sand to this point, place the iron over the die resting upon the sand, now use a short iron ring tapering, and cover the die, say $1\frac{1}{2}$ inches, with a room of $\frac{1}{2}$ inch around, prevent the lead from running out between the ring and plate, by placing sand firmly around—pour the lead into the ring and you have a perfect female die.

There is some judgment required as to the temperature of the melted metal, which should never be hot enough to ignite paper. We would advise the universal adoption of one alloy so as to overcome the difficulties of shrinkage by a long experience in its use, and thus be able to refer your troubles to their proper source, much annoyance will thus be avoided, and considerable progress made; but in using differently composed dies you will encounter obstacles the sources of which you cannot trace. In throwing off these hasty remarks, we have been induced to do so, more by the desire of enlisting information from others than from the hope of imparting any thing new or untried to those who are laboring to overcome the disadvantages all dentists must encounter in mechanical work.

